# Additional Personal

**Published:** 1898-06

**Authors:** 


### ADDITIONAL PERSONAL.

Dr. William House, of 1106 Main street, is caring for the practice of Dr. Eugene A. Smith during the latter's absence as major in the 65th regiment. Dr. House will continue Dr. Smith's usual office hours at 831 Ellicott street.

